# Septic shock in older people: a prospective cohort study

**DOI:** 10.1186/1742-4933-10-21

**Published:** 2013-06-06

**Authors:** Fabiano Pinheiro da Silva, Fernando Godinho Zampieri, Denise Frediani Barbeiro, Hermes Vieira Barbeiro, Alessandra Carvalho Goulart, Francisco Torggler Filho, Irineu Tadeu Velasco, Luiz Monteiro da Cruz Neto, Heraldo Possolo de Souza, Marcel Cerqueira César Machado

**Affiliations:** 1 Emergency Medicine Department, University of São Paulo, São Paulo, Brazil

**Keywords:** Ageing, Sepsis, Inflammation

## Abstract

**Background:**

Septic shock is the first cause of death in Intensive Care Units. Despite experimental data showing increased inflammatory response of aged animals following infection, the current accepted hypothesis claims that aged patients are immunocompromised, when compared to young individuals.

**Results:**

Here, we describe a prospective cohort study designed to analyze the immune profile of this population.

**Conclusion:**

Older people are as immunocompetent as the young individual, regarding the cytokines, chemokines and growth factors response to devastating infection.

## Background

Ageing is a growing field of medical research, since this process is strongly related with diseases such as atherosclerosis, Alzheimer’s Disease, type 2 diabetes and other main causes of death worldwide. Older age is associated with chronically elevated circulating levels of inflammatory markers, such as tumor necrosis factor (TNFα), IL-1β, IL-6 and C-reactive protein (CRP) [1]. Immunosenescence seems to influence many components of the immune system. Profound changes in T cell function have been described in older individuals [2,3], especially in Th17 and regulatory T cells activity [2,4]. Regarding innate immunity, age-associated alterations have been described in macrophages, natural-killer and polymorphonuclear cells [5,6]. Discrepant results, however, are frequently observed [7]. While some authors have detected an increase in cytokine production [8], for example, others have described opposing results [9].

Sepsis is a disease of older people. Indeed, 60% of all sepsis events and 80% of septic deaths occur in individuals over 65 years-old [10]. The incidence increases exponentially with age and older age is an independent risk factor for mortality among adults hospitalized with sepsis [11]. A recent prospective cohort study of patients hospitalized with pneumonia-induced sepsis found an age-related increase in in-hospital, 90-day and 1-year post-discharge mortality [12].

The reason for the higher susceptibility to infection and the higher risk of severe sepsis and mortality in older adults, however, remain unclear [13]. Here, we investigate the cytokine profile of aged individuals compared to young adults, during the development of overwhelming infection, in order to characterize the immune status of this population.

## Results

We included 73 patients in this study protocol (44 patients admitted in the ICU for non-infectious reasons and 29 admitted for septic shock) between October 2010 and October 2012. In our study, patients in the ICU control and septic shock groups showed similar profile when young and aged patients were compared. Sex distribution was similar in both groups. Regarding the population characteristics (Table 1), elderly patients in the septic shock group showed worst renal function than young patients in the same group (p = 0.017), as accessed by creatinin plasma levels. Both young and aged patients showed similar sodium, potassium, magnesium, phosphate and chloride plasma levels, whether in the ICU or septic shock group. Calcium, a marker of severity of disease [14], was significantly lower in aged septic individuals than in aged controls (p = 0.043), but not than in young septic patients (p = 0.294). Hemoglobin (Hb) and hematocrit (Ht) levels significantly decreased in young septic patients compared to controls, but not in aged patients. Leukocytes and polymorponuclear cells (PMNs) significantly rose in young septic group compared to controls, but not in the elderly group. Platelets levels were similar in all study groups. Albumin significantly decreased in septic shock when compared to ICU controls, but young and aged patients showed similar albumin levels (p = 0.708). Lactate significantly rose in young, but not in the elderly, when patients in septic shock were compared to controls. The same profile was found for C-reactive protein levels. SOFA score and mortality were similar for the young and the older age septic groups (p = 0.517 and 0.986).

**Table 1:** Population characteristics

	**Young**			**Elderly**			**Group A vs. B**
	**Control n=36**	**Septic shock (group A) n=21**	**p-value**	**Control n=8**	**Septic shock (group B) n=8**	**p-value**	**p-value**
*age (yrs)*	45 ± 11	40 ± 13	0.154	73 ± 6	75 ± 9	0.666	0.000
*male sex*	38%	50	0.454	50	62%	0.766	
*creatinin (mg/dl)*	1.50 ± 2.73	1.04 ± 1.24	0.523	1.50 ± 1.60	2.62 ± 1.40	0.173	0.017
*sodium (meq/dl)*	142 ± 11	139 ± 9	0.133	141 ± 6	146 ± 18	0.454	0.283
*potassium (meq/dl)*	3.52 ± 0.84	3.52 ± 1.16	0.988	3.62 ± 0.74	4.25 ± 0.70	0.122	0.055
*magnesium (mg/dl)*	1.44 ± 0.55	1.19 ± 0.51	0.147	1.38 ± 0.51	1.63 ± 0.51	0.302	0.064
*calcium (mg/dl)*	4.77 ± 0.86	4.42 ± 0.74	0.100	4.75 ± 0.46	4.12 ± 0.64	0.043	0.294
*phosphate (mg/dl)*	3.03 ± 1.15	3.86 ± 2.47	0.073	3.75 ± 1.66	4.38 ± 1.92	0.482	0.577
*chloride (mg/dl)*	107 ± 12	105 ± 7	0.527	108 ± 10	104 ± 6	0.394	0.703
*hemoglobin (g/dl)*	12 ± 2.0	10 ± 2.2	0.011	10 ± 2.0	10 ± 2.2	0.913	0.939
*hematocrit (g/dl)*	36 ± 6	30 ± 6	0.010	31 ± 6	32 ± 6	1.000	0.769
*leucocytes (/mm3)*	10891 ± 4386	15569 ± 13341	0.046	17830 ± 23033	11863 ± 6020	0.499	0.314
*PMN cells (/mm3)*	8778 ± 4003	12406 ± 10710	0.061	7518 ± 3917	10446 ± 5632	0.231	0.529
*platelets (/mm3)*	235833 ± 136039	224571 ± 171250	0.769	196500 ± 86663	152750 ± 69134	0.267	0.119
*albumin (g/dl)*	2.83 ± 0.81	2.04 ± 0.74	0.000	2.62 ± 0.51	2.12 ± 0.35	0.039	0.708
*lactate (g/dl)*	12.75 ± 6.83	31.61 ± 35.91	0.001	11.25 ± 4.71	19.12 ± 9.32	0.311	0.154
*CRP (g/dl)*	50 ± 76	139 ± 129	0.003	56 ± 73	96 ± 162	0.494	0.939
*SOFA score*	3.50 ± 3.12	9.14 ± 4.13	0.000	4.13 ± 3.22	9.25 ± 2.96	0.002	0.517
*ICU mortality*	11%	47%	0.003	37.5%	50%	0.628	0.939

Cytokines profile showed activation of innate immunity and polyclonal activation of T cells in the young during septic shock, characterized by a rise in TNFα, IL-1β, IL-6, IL-4, IL-17 and interferon-γ when compared to the ICU control group. No differences, however, could be detected when the young in septic shock (group A) was compared to the elderly in septic shock (group B) (Table 2). Chemokines and growth factors also showed similar values when the elderly was compared to young subjects (Table 3).

**Table 2:** Cytokines profile

	**Young**			**Elderly**			**Group A vs. B**
	**Control n=36**	**Septic shock (group A) n=21**	**p-value**	**Control n=8**	**Septic shock (group B) n=8**	**p-value**	**p-value**
*IL-1α*	31 ± 42	100 ± 82	0.000	27 ± 27	75 ± 82	0.270	0.242
*IL-1β*	2.50 ± 2.93	7.09 ± 7.11	0.007	1.12 ± 1.35	4.37 ± 6.98	0.480	0.148
*IL-1 RA*	36 ± 47	192 ± 295	0.001	31 ± 33	145 ± 292	0.114	0.317
*IL-2*	9.00 ± 6.74	13 ± 10	0.121	4.12 ± 2.10	9.62 ± 5.52	0.023	0.508
*IL-3*	2.08 ± 2.44	2.61 ± 3.33	0.849	0.62 ± 0.51	2.25 ± 2.54	0.070	0.899
*IL-4*	8.55 ± 10.60	32 ± 48	0.007	3.87 ± 4.91	16.00 ± 22.35	0.093	0.334
*IL-5*	2.19 ± 3.24	7.19 ± 9.26	0.010	0.50 ± 1.06	2.87 ± 3.44	0.082	0.208
*IL-6*	37 ± 63	1641 ± 3195	0.000	32 ± 39	1666 ± 3590	0.036	0.884
*IL-7*	11 ± 8.26	40 ± 39	0.000	5.62 ± 3.46	34.75 ± 48.70	0.002	0.393
*IL-8*	29 ± 25	806 ± 2819	0.000	26.25 ± 22.24	1695 ± 4602	0.016	0.354
*IL-9*	2.08 ± 2.37	4.52 ± 5.06	0.044	0.50 ± 1.06	4.25 ± 6.11	0.043	0.489
*IL-10*	12 ± 16	626 ± 2195	0.000	38 ± 78	480 ± 1079	0.012	0.575
*IL-12p40*	39 ± 44	111 ± 80	0.000	31 ± 34	141 ± 214	0.206	0.464
*IL-12p70*	7.19 ± 4.79	20.61 ± 20.09	0.002	4.50 ± 2.72	10.50 ± 8.79	0.423	0.195
*IL-13*	4.86 ± 6.51	14.90 ± 32.48	0.119	1.37 ± 1.06	10.87 ± 16.12	0.097	0.583
*IL-15*	7.38 ± 6.23	20.52 ± 25.30	0.001	4.00 ± 3.20	16.62 ± 18.92	0.011	0.558
*IL-17*	2.88 ± 2.43	8.66 ± 6.31	0.000	1.87 ± 1.45	6.87 ± 5.46	0.018	0.493
*IFNα*	67 ± 42	110 ± 58	0.000	195 ± 402	78 ± 38	0.270	0.150
*IFNy*	7.83 ± 4.45	40.09 ± 52.41	0.000	6.25 ± 5.67	30.87 ± 36.56	0.013	0.574
*TFNα*	19 ± 21	111 ± 207	0.000	23 ± 20	81 ± 102	0.016	0.770

**Table 3:** Chemokines and growth factors profile

	**Young**			**Elderly**			**Group A vs. B**
	**Control n=36**	**Septic shock (group A) n=21**	**p-value**	**Control n=8**	**Septic shock (group B) n=8**	**p-value**	**p-value**
*EGF*	266 ± 212	212 ± 182	0.264	192 ± 80	250 ± 213	0.600	0.696
*FGF2*	122 ± 62	133 ± 55	0.301	113 ± 45	153 ± 67	0.344	0.575
*FITL3*	6.27 ± 11.4	18 ± 165	0.000	6.25 ± 14.04	16 ± 27	0.235	0.104
*FRAKTALINE*	167 ± 81	573 ± 858	0.001	120 ± 73	296 ± 273	0.027	0.251
*GCSF*	156 ± 196	3662 ± 7100	0.001	104 ± 106	3226 ± 7166	0.093	0.922
*GMCSF*	32 ± 21	125 ± 284	0.000	48 ± 65	54 ± 43	0.208	0.241
*GRO*	1958 ± 1642	2644 ± 3934	0.772	1733 ± 884	4074 ± 4718	0.753	0.435
*MCP-1*	610 ± 912	2806 ± 4334	0.002	916 ± 1129	3237 ± 4792	0.171	1.0
*MCP-3*	24 ± 13	58 ± 44	0.001	11.62 ± 5.20	32 ± 25	0.023	0.130
*MDC*	817 ± 719	714 ± 471	0.869	745 ± 350	625 ± 265	0.208	0.527
*MIP-1*	23 ± 22	50 ± 65	0.069	322 ± 871	39 ± 25	0.127	0.696
*MIP-1*	42 ± 25	102 ± 83	0.000	35 ± 31	94 ± 60	0.027	0.678
*VEGF*	227 ± 306	310 ± 239	0.003	143 ± 115	392 ± 387	0.021	0.678

## Discussion

Older age has been widely characterized as a pro-inflammatory state [15]. Animal models of infection also support that older subjects have higher circulating levels of inflammatory markers. Tateda et al. have shown that aged mice exhibit higher TNFα, IL-1β and IL-6 levels than young mice after intraperitoneal injection of LPS [16]. Using the cecal ligation and puncture model (CLP), Turnbull et al. also found similar results [17]. The underlying mechanism of the vulnerability to infections of the elderly has been ascribed to the breakdown of anatomic barriers, underlying illnesses or the declining capacity of the immune system [18], while latent infections such as cytomegalovirus (CMV) may act as chronic stimuli of the inflammatory system [19,20]. Persistent inflammation has been shown to increase the risk of bacterial invasion in rodents by reducing neutrophil recruitment and bacterial clearance [21,22]. Turnbull et al. proposed that macrophages of aged mice may be relatively activated, or primed, for cytokine production prior to LPS challenge. There have been, however, several conflicting reports, when in vitro production of cytokines by aged cells is investigated [23-26]. In our study, we were also unable to find any cytokine, chemokine or growth factor that could serve to better explain the reasons why this population shows increased susceptibility and mortality to septic shock. Our results show, moreover, that leukocytosis, CRP and lactate are good markers of infection to young patients, but not to the aged one. We suggest that the increased mortality of the elderly described by others may underlie on the function of non-immune cells. Disruption of epithelial barriers, malfunction of ciliary cells, malabsorption, malnutrition, polimorphisms, deficits of cognition and associated comorbities, in our opinion, may play a pivotal role. Interestingly, impaired sympathetic response has been observed as a partial explanation for several modifications that occur during ageing [27], including increased mortality in septic shock [28].

Sepsis has challenged the medical community for decades. After the negative results of innumerous clinical trials, when researchers insisted that the disease could be controlled by inhibition of the pro-inflammatory response, a new hypothesis emerged. In 1996, Roger Bone proposed that early after the explosive inflammatory response that characterizes sepsis, a compensatory antagonistic response syndrome (CARS) would arise [29], explaining why anti-inflammatory drugs do not benefit septic patients and even further increase mortality. It is important to state that our study looks at the immune profile at one point in time. It is possible that immune profiles diverge later in disease. This point is important later when we talk about CARS, since CARS may still exist later in the disease course.

Actually, sepsis is considered a heterogenic disease, being very difficult to reproduce in animal models. Indeed, at the present time most of the researchers believe that a systemic inflammatory response syndrome (SIRS) prevails in young patients, while CARS dominates most of the septic inflammatory response of older people. In aged patients, thus, sepsis would be characterized by a dominant anti-inflammatory response, an immunocompromissed state [30]. Clinical trials, however, have not confirmed this hypothesis yet. Our study points out that the elderly has an efficient immune profile, very similar to the young patient.

Our study has limitations. The major limitation is the sample size. A recent clinical study, however, was unable to find any age-related difference in inflammatory and cell surface markers in patients hospitalized for pneumonia [12]. Similarly, Kelly et al. found no differences in the cytokine profile of young, older and very elderly patient with community-acquired pneumonia [31]. We believe that our data brings important information and should be further explored in larger studies. Once confirmed, studies of immunosenescence should focus on defects in phagocytosis, production of reactive oxygen species, immunoglobulins and other cell functions that were not addressed by the present study.

## Conclusions

Our study describes that aged individuals display a competent immune profile, regarding the secretion of cytokines, chemokines and growth factors. Further research is warranted to explain the molecular mechanisms why aged individuals develop higher rates of infection and might display worse outcomes, a relevant topic on immune-inflammatory responses in ageing. Here, we point out that the results in animal models diverge from clinical research and argue against the current concept that sepsis in older people is characterized by CARS, a massive counter-antagonistic response to systemic inflammation. Larger studies are necessary to confirm our data and explore other aspects of immunity in this population.

## Methods

### Study design

The current study was a prospective cohort, conducted in one of the Hospital das Clinicas Intensive Care Units (University of Sao Paulo, Brazil). Surgical patients, trauma and coronary syndromes are usually admitted at other ICUs from our hospital, what makes our population very homogeneous. Indeed, the five following reasons account for more than 90% of the ICU admissions included in this study: sepsis, stroke, altered level of consciousness, pulmonary edema and asthma/COPD. Patients less than 18 years old, pregnant, with disseminated malignancies or receiving chemotherapy, HIV-positive, advanced hepatic disease, in end-of-life conditions and those that refused to participate in this study were excluded. The remaining patients were divided in two groups: 1) ICU control (patients admitted to the ICU for non-infectious causes); 2) septic shock group (patients admitted to the ICU for septic shock or that developed septic shock during the ICU stay). 90% of data from septic elderly were taken on hospital day 1 compared to 90% from elderly controls. Each group was divided in two sub-groups: under 65 years-old (young individuals) and above 65 years-old (aged individuals). Data and blood samples were collected at patient’s admission or when the diagnosis of severe sepsis or septic shock was established by the medical staff. SIRS, Severe sepsis and septic shock were defined, according to the consensus proposed in 1992 [32]. The protocol was approved by Hospital das Clinicas Ethical Committee and patients (or their close relatives) received detailed explanations and provided written consent to be included in the study protocol.

### Statistical analysis

A descriptive analysis of the population (age, sex, electrolytes, SOFA score and mortality) was performed. SOFA score assesses mortality in the critically ill. Cytokines were analyzed by Miliplex technology (Merck, Genese diagnostics), a multiplex method for cytokines analysis. Continuous variables were analyzed by using Student’s t test or Mann–Whitney test, as appropriated. Categorical variables were analyzed by using chi-square test. Results were reported as mean ± standard deviation. All analysis performed used SPSS 19.0 software. A *p* value of .05 was considered to be statistically significant.

## Competing interests

The authors have no financial or ethical conflicts of interest.

## Authors’ contributions

FPS designed the experiments and wrote the paper, FGZ collected the samples, DFB and HVB performed the experiments, ACG and FTF designed the data bank. The remaining authors supervised the study. All authors revised the first draft. All authors read and approved the final manuscript.
